# CP-CHARM: segmentation-free image classification made accessible

**DOI:** 10.1186/s12859-016-0895-y

**Published:** 2016-01-27

**Authors:** Virginie Uhlmann, Shantanu Singh, Anne E. Carpenter

**Affiliations:** Biomedical Imaging Group, Swiss Federal Institute of Technology (EPFL), Lausanne, Switzerland; Imaging Platform, Broad Institute of Harvard and MIT, Cambridge, MA USA

**Keywords:** Image classification, Biological imaging, Image features, Segmentation-free analysis, High-dimensional classification

## Abstract

**Background:**

Automated classification using machine learning often relies on features derived from segmenting individual objects, which can be difficult to automate. WND-CHARM is a previously developed classification algorithm in which features are computed on the whole image, thereby avoiding the need for segmentation. The algorithm obtained encouraging results but requires considerable computational expertise to execute. Furthermore, some benchmark sets have been shown to be subject to confounding artifacts that overestimate classification accuracy.

**Results:**

We developed CP-CHARM, a user-friendly image-based classification algorithm inspired by WND-CHARM in (i) its ability to capture a wide variety of morphological aspects of the image, and (ii) the absence of requirement for segmentation. In order to make such an image-based classification method easily accessible to the biological research community, CP-CHARM relies on the widely-used open-source image analysis software CellProfiler for feature extraction. To validate our method, we reproduced WND-CHARM’s results and ensured that CP-CHARM obtained comparable performance. We then successfully applied our approach on cell-based assay data and on tissue images. We designed these new training and test sets to reduce the effect of batch-related artifacts.

**Conclusions:**

The proposed method preserves the strengths of WND-CHARM - it extracts a wide variety of morphological features directly on whole images thereby avoiding the need for cell segmentation, but additionally, it makes the methods easily accessible for researchers without computational expertise by implementing them as a CellProfiler pipeline. It has been demonstrated to perform well on a wide range of bioimage classification problems, including on new datasets that have been carefully selected and annotated to minimize batch effects. This provides for the first time a realistic and reliable assessment of the whole image classification strategy.

**Electronic supplementary material:**

The online version of this article (doi:10.1186/s12859-016-0895-y) contains supplementary material, which is available to authorized users.

## Background

Images are essential in biomedical research. As large amounts of data are routinely acquired, automated image analysis has become unavoidable. A variety of important biomedical research problems therefore rely on supervised image classification, and automated image classification using machine learning is increasingly used in many biomedical research laboratories in order to extract information from the rapidly expanding amount of acquired data. A common paradigm when classifying biological images is to first segment the objects in the image to isolate them from the background and then to extract features from them. Unfortunately, configuring algorithms for accurate object segmentation is often not trivial and is likely to be the bottleneck that constrains the quality of an analysis, as inaccurate segmentation yields inaccurate results. In addition, the design of a custom segmentation algorithm often requires significant expertise. Finally, because of the absence of widely generalizable segmentation methods, reusing existing algorithms for a new task is often tedious, time-consuming and sometimes even impossible.

To address this problem, it has been proposed that classification can also be performed without segmenting regions of interest, by computing a large number of features on the whole image and then selecting the most discriminative ones. Segmentation-free image classification using whole-image features has already been widely used in computer vision, especially for image databases [1, 2]. It is however less popular for bioimage analysis, where segmentation remains the most common paradigm. Recently, several whole image-based methods have been proposed for particular biological imaging classification problems such as protein subcellular localization [3–5] and immunostaining pattern identification [6]. Among these, some more general-purpose and customizable software for high-throughput screening have been released [7, 8]. One such approach, named WND-CHARM (Weighted Neighbor Distances using a Compound Hierarchy of Algorithms Representing Morphology, [9]), obtained promising results on a wide range of different datasets, including biological ones [10, 11]. It appeared as particularly interesting from its adaptability to a variety of classification problems. Concerns were however later raised because in some of these data sets, training and test images were taken from the same experimental batch and reasonable classification accuracy could be obtained using background regions of the images or images where the biological entities of interest had been replaced with featureless boxes [12]. This indicated that the classification signal actually derived from unanticipated information due to some systematic effect on sample preparation or image acquisition that can distinguish classes, leading to results that were not representative of the actual performance of the method. As an example of this situation, consider a case where positive controls are imaged on a single day and negative controls are imaged the following day. Even slight, subtle changes in experimental conditions (such as the brightness of the lamp on the microscope, which changes depending on how long it has been warmed up) could be present such that the two image classes are distinguishable. A classifier trained and tested on these data could then separate classes very efficiently based on illumination differences, which have no biological meaning. As this effect is spread over the whole image, segmenting the biological objects of interest does not solve the problem. We refer to these effects as *batch-related* or *global* artifacts through the paper.

Solutions to avoid these artifacts have been proposed, which include ensuring that the training and test sets are collected in a way that systematic effects in the images cannot enable spurious discernment among the classes or relying on image metadata to exclude repetition of samples acquired in similar conditions [12]. Alternatively, “manual” assessment of the quality of results is possible, for instance by removing biologically relevant content and observing the effect on classification accuracy, as proposed by [12]. Similar findings have been reported by [13] in the particular case of subcellular protein localization. It was observed that relying on a single type of protein for each location class yielded overoptimistic classification results that did not generalize well when considering multiple proteins in each location class. Ensuring the same protein never to be present both in the training and test set was demonstrated as a good way to avoid this kind of bias.

In this study, we developed and tested a segmentation-free algorithm that is inspired by WND-CHARM, has the same advantages, and is more readily usable by those without computational expertise. Our particular interest in focusing on WND-CHARM is driven by its ability to perform on a wide range of different classification problems as well as its algorithmic simplicity. Thus, it is a simpler yet efficient alternative to more complete and complex tools such as BIOCAT [8]. Our method can hence be seen as a variation of WND-CHARM which benefits from enhanced user-friendliness and holds the same application potential. Being designed as a command-line tool, WND-CHARM unfortunately requires considerable computational expertise to execute. Therefore, our motivation for the design of CP-CHARM is two-fold. First, we followed the steps of WND-CHARM in order to mimic its global algorithmic workflow and reproduce its capacity to be effective on many types of data. Then, we adapted all of these steps to rely on standard and widely used methods so as to make the approach comprehensive and user-friendly while also paying attention to execution time. In particular, feature extraction is performed in CellProfiler (CP, [14–16]), an open-source software that has already been widely adopted by the biology community. Although methods that do not require segmentation have achieved state-of-the-art classification results, they have not previously been available in existing GUI frameworks such as CellProfiler. CellProfiler is a unique tool that allows scientists to perform advanced image analysis on large image sets even in the absence of extensive computer programming skills. Image processing steps are organized in a user-friendly “pipeline”, and the various operations on images can be performed by simply adding “modules” to the pipeline. Its simple interface makes the software easy to use, as the various parameters required by the different image analysis algorithms are listed and described in a comprehensive manner. It has been independently rated as the most feature-filled and user-friendly software in its class [17].

We first validated our approach by reproducing WND-CHARM’s original results and ensuring that CP-CHARM could achieve similar performance. Then, we illustrated the usability of our approach on several kinds of biological datasets, namely high-throughput cell-based assay data freely available from the Broad Bioimage Benchmark Collection (BBBC, [18]) and tissue images from the Human Protein Atlas (HPA, [19]). In our experiments, we carefully conducted the validation to avoid allowing systematic artifacts to yield falsely optimistic classification accuracy. As such effects could appear when taking multiple images from the same biological “batch” (for instance in cases where images are actually sub-images of a larger image, or where several images are acquired from the same well), we relied on metadata to make sure not to include a given batch’s images in both the training and test sets. In this way, we ensured that image artifacts unique to each batch (if present) would not yield accurate classification of that batch, irrespective of the actual biological content/phenotype in the image, hence reducing the risk of misleading classification results that can be attributed to global image artifacts. Although not implemented in this paper, the solution proposed by [13] for reducing classification bias could further improve the generalization capabilities of CP-CHARM for the particular classification experiments where different subpopulations belonging to the same class can be identified and hence separated for the training and the testing phase. As a summary, our main contribution in this work is to provide a user-friendly whole-image-based classification method inspired by WND-CHARM, to demonstrate that it yields similar performance, to characterize some of its behavior on biological data sets, and to attract researchers’ attention to batch effects when relying on such approaches.

The paper is organized as follows: in the ‘Implementation’ section, we first recall the structure of the WND-CHARM algorithm and then present and motivate the construction of CP-CHARM. In the ‘Experiments’ section, we present the methodology for the different validation experiments we performed and introduce the additional datasets we used to further test the applicability of CP-CHARM. Experimental results are presented and discussed in the the ‘Results and discussion’ section, followed by concluding remarks.

## Implementation

### Review of WND-CHARM

WND-CHARM is a four-step algorithm. A thorough mathematical description of each step can be found in [10]. First, features are extracted to build the *CHARM* (Compound Hierarchy of Algorithms Representing Morphology) vector composed of 1025 elements (see Additional file 1: Section 1), which is the main novelty of the algorithm. The second step is a selection of the elements of the feature vector to reduce the dimensionality of the feature space. A weight based on the Fisher Discriminant score is computed for each feature. These features are then ranked, and the features corresponding to the 15 *%* top-ranked weights are used to train a classifier based on a weighted *k*-nearest-neighbors-like algorithm. The difference between this Weighted Neighbor Distance (*WND*) method and the traditional *k*-nearest-neighbors approach is that, given a feature vector input, WND computes its weighted distance to all elements in the training set. Every training set element therefore plays a role in the actual classification, while being weighted by some measure of its information content, here based on the ratio between intra- and inter-class variance. Conversely, in *k*-nearest-neighbors only *k* elements from each class equally influence the classification result. Finally, the performance of the classifier is validated using a custom method where 25 *%* of the data in each class sampled at random are saved to constitute the test set, while the remaining 75 *%* in each class is used as a training set. We refer to this method as *lone 4-fold cross-validation* through the rest of the paper.

### Design of CP-CHARM

In order to build CP-CHARM, we independently investigated and adapted each of these four steps, namely feature extraction, dimensionality reduction, classification, and validation. CP-CHARM therefore follows the basic concept of WND-CHARM while relying on more common and readily usable tools. A global view of the proposed algorithm is depicted in Fig. 1. Its workflow is summarized in the following.

1. **Feature extraction:** A total of 953 features are extracted on a whole-image basis using CellProfiler.

**Fig. 1:**
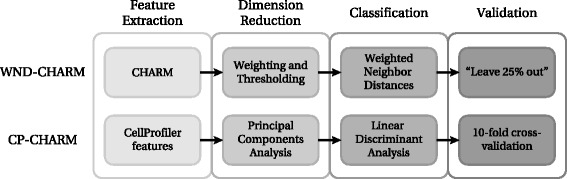
Comparison between WND-CHARM [10] and the CP-CHARM algorithm presented in this paper. The overall construction of the algorithm is retained, but individual operations have been modified
2. **Dimension reduction:** Features are projected in principal components space, and a subset of principal components analysis (PCA) vectors is retained such that 98 *%* of the variance present in the original data distribution is conserved.
3. **Classification:** Linear Discriminant Analysis (LDA) is used to classify the selected PCA-transformed feature vectors.
4. **Validation:** The classifier’s performance is assessed with 10-fold cross-validation.

#### Feature extraction

Regarding feature extraction, we hypothesize that it is more the image-based aspect rather than the actual features extracted that grant WND-CHARM its analysis power. We thoroughly studied the CHARM vector composition as well as the dimension reduction and classification methods in [20]. We explored the feature vector composition, to study the impact and discriminative power of each element (or rather “groups” of elements) in the original CHARM vector so as to get insight on how to construct a feature vector that could achieve similar classification performance. To do this, we first split the CHARM vector into eight feature “groups” (moments features, texture features, edge features, etc.), and then into three feature “levels” (features extracted on the original image, on transform of the image, and on transforms of transforms of the image). The seven datasets from WND-CHARM’s reference suite were then classified by excluding each “group” and “level” at the time, in order to identify which “group” or “level” could best explain classification performance. It appeared that, over the seven considered datasets, no single feature “group” could be identified as consistently being more discriminative than the others (Additional file 1: Section 4, Figure S3). Similarly, although features from the first “level” (i.e., extracted directly from the original image) explained most of the good classification results, the other feature “levels” could not totally be discarded in order to reach WND-CHARM’s performance (Additional file 1: Section 4, Figure S4). Finally, the composition of the top 15 *%* features subset used by WND after feature selection was also studied. There again, it appeared that features from all “groups” and “levels” played a role among the different datasets (Additional file 1: Section 4, Figure S5). More details on these experiments can be found in [20]. We thus drew the conclusion that no isolated feature group seemed to be sufficient to explain the good performance on diverse datasets. All the groups and levels appeared to be important, as class separation was achieved using different morphological aspects depending on the nature of the images. A multi-groups and multi-level vector like CHARM therefore seemed to be an efficient solution to ensure good generalization capabilities, giving additional support for the construction of our CellProfiler feature vector. In this setting, another whole-image-based feature vector composed of judiciously chosen elements might give equally good results. We therefore retained the global construction of the CHARM vector relying on feature groups (high contrast features, polynomial decompositions, pixel statistics and texture measurements) and image levels (transforms and compound transforms), but did not necessarily select the same individual measurements inside each group. Following our goal to develop a flexible and user-friendly tool, we designed the feature extraction step as a CellProfiler pipeline composed of different modules for the extraction of the many measurements required to build the feature vector (Additional file 1: Section 2). In this situation, the parameters of each module are visible to the user and can hence easily be fine tuned to refine the analysis depending on the dataset. The content of the resulting CHARM-like CellProfiler feature vector is depicted in Table 1. While mostly similar to the CHARM vector in the way it is built, it does not contain the exact same elements. We note that the set of features present in the CHARM-like vector is likely to capture image information at a variety of scales. The feature vector contains, in particular, features computed on the wavelet transform of the original image. One of the main interest of the wavelet transform is its ability to extract image features from structures at different scales by tuning the number of wavelet scales to be computed. The Gabor features serve the same purpose, and extract information at different scales. Therefore, we have no reason to believe the proposed approach to be only able to discriminate classes based on a limited range of structure size. Image measurements are extracted using pre-existing CellProfiler modules as well as several newly implemented ones. In particular, modules computing histograms, moments, Tamura texture descriptors, as well as modules computing the Fourier, Haar wavelet and discrete Chebyshev transforms have been developed and added to CellProfiler in the context of this work. In these new modules, we relied on functions from the popular Python library *scikit-learn* [21] when possible in order to minimize duplication of code. This strategy, and the resulting vector components, are also reminiscent of the feature set proposed by [22] for the task of classifying protein cellular localization.

**Table 1:** Composition of the CHARM-inspired CellProfiler feature vector. The groups and levels construction of CHARM was recreated, although the final measurement set is not identical

High-contrast features			Polynomial decompositions		Pixel statistics		Textures	
Edge	Gabor	Image	Chebyshev	Chebyshev-	Moments ^∗^ (4)	Multiscale	Haralick	Tamura
statistics (4)	features ^∗^ (2)	statistics (15)	statistics (32)	Fourier		histogram ^∗^ (24)	textures ^∗^ (104)	textures ^∗^ (6)
				Statistics (32)				
Mean	Gabor features computed at four angles	Max ^∗^	32-bins histogram of the 400 coefficients of the Chebyshev transform of the image	Modulus of the complex coefficients of the Fourier transform of the Chebyshev transform of the image	Mean (1st)	3-bins histogram	Statistics based on the co-occurence matrix of the image	Contrast
Max		Mean ^∗^			Variance (2nd)			Coarseness
Variance		Percent minimal ^∗^				5-bins histogram		Directionality
Number of edge pixels					Skewness (3rd)			3-bins histogram of coarseness
		Percent maximal ^∗^				7-bins histogram		
					Kurtosis (4th)			
		Variance ^∗^				9-bins histogram		
		Total intensity ^∗^						
		Mean intensity after thresholding						
		Variance on thresholded image						
		Number of pixels above threshold						

^*^denotes features extracted on higher image levels, namely on the original image, on its Wavelet transform, on its Chebyshev transform, on its Fourier transform, on the Wavelet transform of its Fourier transform, and on the Chebyshev transform of its Fourier transform

#### Dimensionality reduction

As in WND-CHARM [23], we followed the strategy of computing a large set of features and subsequently applying dimension reduction. In this way, the algorithm can automatically adapt to a given dataset through the dimension reduction step. We replaced the custom dimensionality reduction method of WND-CHARM with a commonly-used and mathematically well-characterized algorithm. CP-CHARM therefore relies on Principal Components Analysis (PCA, [24]) for dimension reduction. The choice of PCA for feature selection brings several advantages over the weighting and subsequent thresholding used in WND-CHARM. First, the method is more standard. Second, its underlying parameters can directly be related to the data distribution, while the threshold value in WND-CHARM is purely empirical. Moreover, the dimension reduction method used in WND-CHARM that selects 15 *%* of the 1025 computed features is unstable and does not account for possible redundancy. We assessed the stability of the feature selection algorithm by computing the Tanimoto distance between the set of remaining features after selection over 100 classification runs on the same data. There, we observed that the selected feature set is unstable (i.e., different sets of features are selected over different runs, see Additional file 1: Section 4, Figure S6). We assessed redundancy by identifying selected features and trying to classify the same datasets by removing some of these features from the feature vector. We found that this did not always affect classification accuracy (as can be seen by comparing Figures 3 and 5 in the Additional file 1: Section 4). Therefore, the feature set selected by WND-CHARM’s custom dimension reduction method seems to exhibit a fair amount of redundancy. Using PCA alleviates this issue, as it provides an orthogonal basis that is non-redundant as well as effective in separating the classes. By doing so, we preserve information that is otherwise lost in WND-CHARM when 85 *%* of the features are discarded. We note that there are other algorithms such as mRMR [25] that address the same problem of feature redundancy. Finally, by varying the feature reduction method/classifier pair, we observed that methods allowing for nonlinearities (such as kernel SVM) did not improve classification performance (Additional file 1: Section 4, Figure S7). We therefore believe that for the datasets tested, linearly separability is a reasonable assumption in the CP-CHARM feature space. Many other feature reduction procedures have been studied for the problem of protein location classification from image-based features [26] and could also have been selected. Further, regularized LDA [27, 28] is another alternative to performing PCA prior to LDA, an approach we have not explored in our experiments. We finally opted for PCA for four main reasons: it is not too computationally demanding, it requires little parameter tuning, it reduces the risk of selecting noisy variables, and it was observed a posteriori to lead to good classification results on many different classification problems. In our implementation, the amount of information (variance) to be preserved when performing PCA can be tuned by the user. It is set by default to 0.98 (98 %).

#### Classification

Our goal was to provide a user-friendly, easy to tune and general-purpose method for classification. We chose Linear Discriminant Analysis (LDA, [29, 30]) as classification method for three principal reasons. First, it has the advantage of being more standard than WND. In addition, the way class separation is determined in LDA is closely related to the Fisher Discriminant score, retaining the initial idea present in WND-CHARM. Finally, we compared the performance of several popular classifiers (PCA-LDA, penalized LDA, radial basis functions support vector machines (SVM), linear SVM, and random forests) using the caret package for R [31] on the test datasets from [10] and [11] and observed PCA-LDA to be the best option as it consistently provided the best trade-off between good classification performance, generalization power and short execution time (Additional file 1: Section 4, Figure S8).

#### Validation

We suspected that the custom validation method used in WND-CHARM might give bad estimates of the true classification efficiency because the classification result is based on only one training set-testing set split of the data. WND-CHARM’s validation method is in fact equivalent to performing only one fold of stratified 4-fold cross-validation: classification accuracy is directly estimated by classifying $\frac {1}{4}$ of the input data after training on the remaining $\frac {3}{4}$. This could be a possible explanation for the larger standard deviations of classification accuracy observed when repeating the classification experiments with WND-CHARM’s validation method. In CP-CHARM, we decided to rely on unstratified *k*-fold cross-validation [32]. In this method, *k* splits of the data are built regardless of their classes. Nothing prevents a split to have no representative of some classes, or conversely to contain all elements of a given class, with the exception of samples being held out to prevent batch effects. In practice, we selected *k*=10. This choice stemmed from the fact that biological datasets are usually composed of a large number of elements. With large sample sizes, the possible number of combinations for each fold becomes large enough that iterations of 10-fold cross-validation do not run a large risk of being duplicates. As this modification had a direct impact on the measure of performance of the method, we performed an extensive validation to assess its effect (see Additional file 1: Section 3). For several datasets, we also compared WND-CHARM’s custom validation method and *k*-fold cross-validation for various values of *k*. We repeated each experiment a hundred times in order to get robust estimates for the median and standard deviation of classification accuracy. The value of *k*=10 was found to be a good choice for obtaining stable estimates of the classification accuracy on all the datasets we considered.

## Experiments

### Validation of the proposed method

Our main goal with CP-CHARM being to propose an equally efficient but more user-friendly version of WND-CHARM, we performed several experiments to ensure that similar performance could be obtained with our algorithm. In order to allow for objective comparison, we re-implemented WND-CHARM in Python, the same programming language as CP-CHARM, following the description of the algorithm given by [10] and the C++ source code from [9]. Our Python version of the WND classifier and its custom validation method is freely available online at [33]. We performed extensive tests to ensure the similarity of results obtained with the original C++ version and our re-implementation [20]. We first investigated the effect of changing WND-CHARM’s validation method while keeping the rest of the algorithmic structure unchanged (Additional file 1: Section 3), and then reproduced WND-CHARM’s published results on two collections of datasets and compared it with the performance of CP-CHARM. Processing time both for WND-CHARM and for our method was on the order of hours, depending on the input image sizes and on the number of elements in the dataset. For both approaches, the most computationally expensive step was feature extraction.

#### Reproduction of WND-CHARM’s results

We based our comparison of CP-CHARM and WND-CHARM on two collections of datasets. The first collection is composed of seven diverse datasets used to assess WND-CHARM’s efficiency in [10]. It is composed of biomedical to face recognition images and aims at showing the algorithm’s usability on a wide range of data. We refer to this collection as *WND-CHARM’s reference suite*. The second collection is the IICBU Biological Image Repository [11], a benchmarking suite for biological image analysis algorithms covering a wide range of biological applications. Results using WND-CHARM on these data are available in [9]. It has been shown [12] that some of the datasets from these two suites, in particular CHO, HeLa, Binucleate and RNAi, contain images likely taken from the same batch. As a consequence, sufficient batch-specific information is present in the images to classify well even in the absence of portions of the images containing cells. Therefore, the computed classification accuracy is overly optimistic and should be taken with caution. We nevertheless included results on these image sets for comparison purposes with WND-CHARM. In all of our experiments, we performed 100 runs of training and testing, each time with different splits of data. The median and standard deviation of the distribution composed of the 100 validation results were taken as measures of classification accuracy.

### Experiments on further biological data

We selected several high-throughput biological image datasets in order to further test the performance of CP-CHARM. In all cases, we relied as much as possible on available metadata to identify images from the same experimental batch. For validation, we made sure not to include images from the same batch in both the training and test sets. In this setting, we limited the chances of successfully classifying a test set element based on common artifact features of one of the training set elements, thus reducing the likelihood of obtaining erroneous classification signal from global artifacts.

#### Broad Bioimage Benchmark Collection image sets

The Broad Bioimage Benchmark Collection (BBBC, [18]) provides several benchmarking sets for image analysis algorithms. The four sets we selected are two-class problems involving a negative and a positive phenotype. All sets are composed of two channel images acquired in 96-well plates, the first channel containing a GFP signal linked to the protein to be observed, and the second containing signal from a stain that labels the cell nucleus.

BBBC013 and BBBC014 feature images from human osteosarcoma cell (U2OS) cytoplasm—nucleus translocation assays. In BBBC013, cells are treated with two different drugs (Wortmannin and LY294002) such that the proteins responsible for the nuclear transport are inhibited and the protein of interest is therefore trapped in the nucleus. Images from the eight positive control wells along with the second highest dose and the negative controls along with the lowest dose of each drugs were selected to constitute the 32 original 640×640 pixel images. The setup of BBBC014 is quite similar but involves a different protein of interest and was composed of 32 1360×1024 pixel images. In both cases, drug-treated cells were considered as positive.

BBBC015 and BBBC016 are two human U2OS cell transfluor assays. In this setting, cells express a receptor and a GFP-tagged protein such that, when stimulated by a particular compound, the receptor triggers a cascade of events leading to the generation of protein vesicles inside the cell. In BBBC015, we selected images of the wells containing the two highest and two lowest compound concentrations, yielding 48 1000×768 pixel images evenly split between positive and negative phenotypes. Built in a similar way, BBBC016 is composed of 18 512×512 pixel images.

**Fig. 2:**
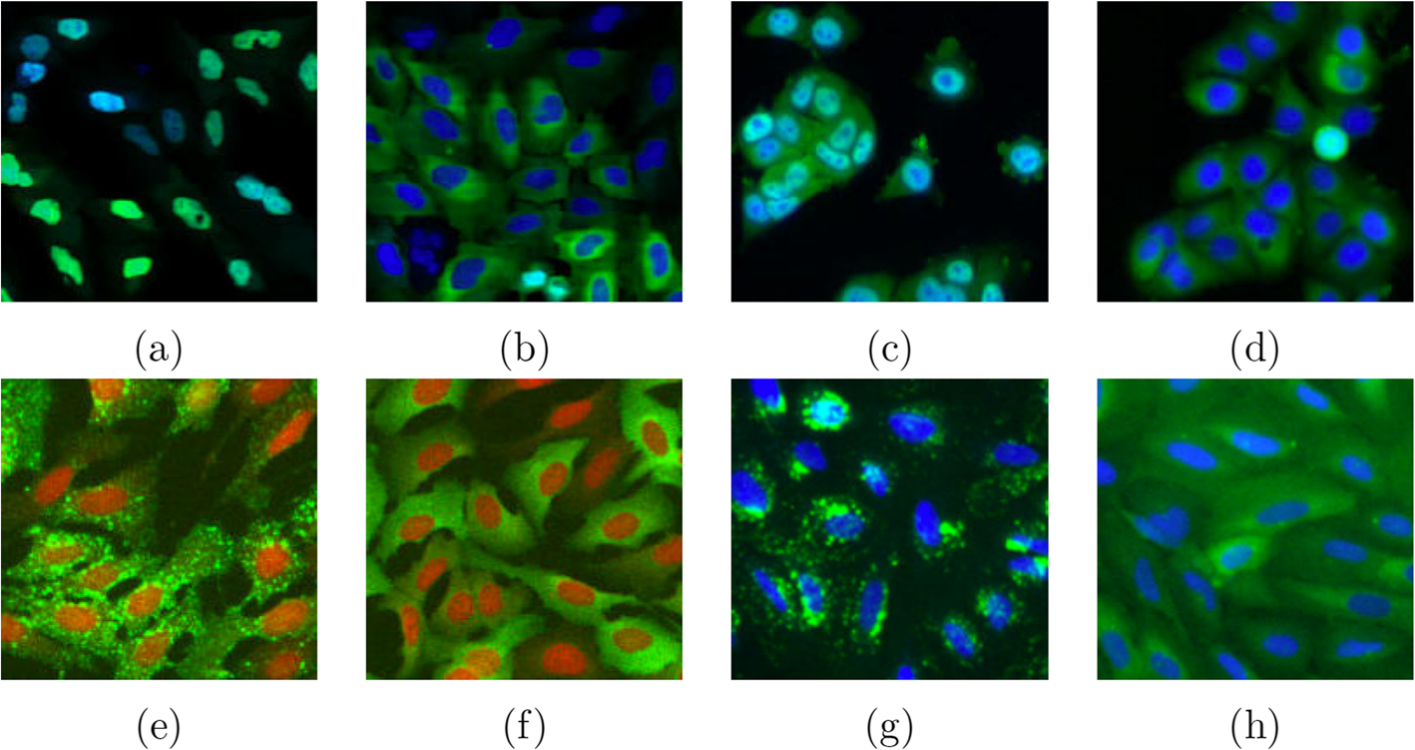
Example of images from each of the tested BBBC datasets. BBBC013: (**a**) positive, (**b**) negative; BBBC014: (**c**) positive, (**d**) negative; BBBC015: (**e**) positive, (**f**) negative; BBBC016: (**g**) positive, (**h**) negative

Examples of images for each class of the four datasets are presented in Fig. 2. In all cases, we excluded the nucleus channel and classified the GFP images only, making the classification challenge more difficult than in [34]. To avoid batch effects, we excluded images from the same well, hence being left with only one field of view per well. For robustness purpose, we performed 100 iterations of training and testing.

#### Human Protein Atlas tissue image set

**Fig. 3:**
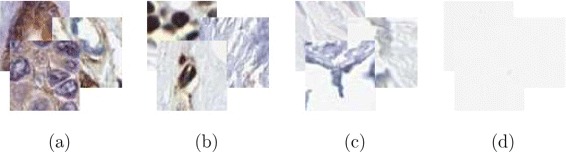
Examples of elements of the four cell compartments classified in the HPA tissue dataset. (**a**) Cytoplasm, (**b**) nuclei, (**c**) connective tissue, and (**d**) background

The tissue dataset compiled by [35] is composed of 1057 64×64 pixel images manually selected from 61,354 patches created by tiling 3000×3000 pixel images of 19 normal and 10 cancerous breast tissue samples from the Human Protein Atlas (HPA, [19]). The classification task is to identify to which of four different subcellular compartments each small image belongs. Samples from each class are presented in Fig. 3. The original color images were composed of two channels, a brown dye targeting a protein of interest and a blue dye nonselectively labeling some cellular components, which were both used for feature extraction. Ground truth was provided as the annotation of the set by a trained expert. In order to reduce the risk of global image artifacts, we grouped patches coming from the same large image such that they would not be split between the test and training sets. Results were obtained from 10 runs of classification experiments.

## Results and discussion

We here present and discuss results of the various experiments we described in the ‘Implementation’ section. First, we show and discuss several comparisons of performance between CP-CHARM and the original WND-CHARM. We then provide classification results using CP-CHARM on the BBBC and tissue datasets.

### Comparison with WND-CHARM performance

We used CP-CHARM to classify datasets from the WND-CHARM’s reference and IICBU suites. We first reproduced published results using our Python implementation of WND-CHARM, which has been shown to give similar results as the published C++ version [20]. Then, we ran CP-CHARM in similar experimental conditions. We obtained results comparable with WND-CHARM, as shown in Table 2. This confirmed that the extracted features, which differ to some degree from those in the original WND-CHARM algorithm, are nonetheless comparably effective at these classification tasks. We were therefore confident that the proposed method was a reliable whole-image based classification algorithm with performance equaling state of the art. We carried out several experiments investigating the feature vector construction, feature reduction method, classifier and validation method in order to identify which aspect of the algorithm improved performance over WND-CHARM [20]. The choice of the feature reduction and classification algorithms appeared to have a greater impact on classification results than the feature vector composition and validation method. The increase in performance using CP-CHARM is therefore most likely not a validation artifact, but a true improvement that can be attributed to the change of the feature reduction and classification methods.

**Table 2:** Classification results on WND CHARM’s reference datasets and IICBU suite

		WND-CHARM^1^		CP-CHARM^2^	
	Dataset	Median	Std Dev.	Median	Std Dev.
WND-CHARM’s reference suite	AT&T	0.97	0.02	0.98	5e-3
	Brodatz	0.91	0.01	0.91	3e-3
	CHO ^∗^	0.93	0.02	0.99	3e-3
	COIL-20	1.00	1e-3	1.00	1e-3
	HeLa ^∗^	0.87	0.09	0.84	4e-3
	Pollen	0.95	0.02	0.95	3e-3
		0.83	0.07	0.84	0.01
IICBU suite	Binucleate ^∗^	1.00	0.01	0.95	0.02
	Liver Aging	0.93	0.03	0.89	4e-3
	Liver Gen. AL	0.98	0.01	0.98	5e-3
	Liver Gen. CR	0.99	0.01	0.99	1e-3
	Lymphoma	0.79	0.04	0.66	0.01
	RNAi ^∗^	0.78	0.04	0.73	0.02
	Terminal Bulb	0.59	0.04	0.55	6e-3

*Note:* 1.0 corresponds to 100 % correct classification

Datasets marked with a star (^∗^) are likely to be subject to global image artifacts. See explanations in text

^1^Using lone 4-fold cross-validation

^2^Using 10-fold cross-validation

We note that, in both data suites, CP-CHARM exhibits less variance in its classification accuracy results. This is not surprising as we used here the original WND-CHARM, including its custom validation technique. The larger variance hence corroborates with the observations drawn from the validation comparison experiment (Additional file 1: Section 3), namely that 10-fold cross-validation is more stable across repetitions of training and testing for the same dataset. We recall that the CHO, HeLa, Binucleate and RNAi datasets have been shown [12] to give good classification results even in the absence of biological information. Although these results should be considered with caution, they nevertheless allow us to compare the kind of performance obtained with CP-CHARM and WND-CHARM. Note that these results compete with state-of-the-art performances [13].

Being confident in the overall approach and implementation of CP-CHARM, we next sought to study its performance on additional datasets. We therefore selected several biological image collections in which inter-class differences are more subtle than in the two test suites and where metadata was available to reduce the impact of global image artifacts.

### Application to additional biological datasets

We found that CP-CHARM yielded good results on the BBBC and the tissue datasets, as observed in Table 3. To assess the significance of these results, we generated randomized versions of each dataset by shuffling class labels. We then classified the shuffled data in order to estimate the median and standard deviation of random classification accuracy. We obtained for BBBC013 0.5±0.05, for BBBC014 0.5±0.05, for BBBC015 0.5±0.04, for BBBC016 0.5±0.07, and for the tissue dataset 0.32±0.02 (tissue dataset has imbalanced classes). Resulting *p*-values allowed us to statistically ensure that CP-CHARM was significantly more efficient than a random classifier.

**Table 3:** CP-CHARM classification results on additional biological datasets

Dataset	Median	Standard deviation
BBBC013	0.99	0.01
BBBC014	0.84	0.03
BBBC015	0.99	8e-3
BBBC016	0.81	0.07
Tissue	0.91	4e-3

*Note:* 1.0 corresponds to 100 % correct classification

Results on the tissue dataset are especially exciting. Tissue image analysis in the context of the HPA project is mostly performed manually so far because segmentation is not well-suited as every part of the image contains information. The good classification accuracy obtained with our algorithm seems to indicate that automated annotation could be possible with a reasonable error rate. Whole-image-based classification algorithms like WND-CHARM fully display their strength in tissue images. On the other hand, cell-based screens like the BBBC image sets are mostly composed of background. One must also keep in mind that whole-image-based analysis sacrifices single cell resolution and is therefore unable to capture phenotypic heteogeneity inside each class. In such situations, cell-based approaches might be equally effective and perhaps even preferred, for example when cell density would be better ignored. More generally, the choice between segmentation or whole-image based approaches shall be driven by considering the effort needed to set up pre-processing/segmenting and the desired results. When segmentation is time-consuming to set up or impossible, whole-image based classification is of particular interest, as even though results might be outperformed by segmentation-based methods, they are obtained rapidly and at minimal hands-on effort.

Good performance of image-based classifiers should be considered with caution as batch-related artifacts might be sufficient to discriminate between the images, leading to results that are not driven primarily by the biological content. It is therefore important to pay particular attention to training and test set composition to reduce batch effects whenever possible, as we did in this study.

## Conclusions

In this paper, our contributions include first the presentation of CP-CHARM, an easy-to-use whole-image-based classification algorithm inspired from the method proposed by [10]. Similar to WND-CHARM, CP-CHARM is generalizable across a wide range of applications, obviating the need for image segmentation. In addition, the proposed approach offers improved user-friendliness, as feature extraction is performed in an easily editable and modular CellProfiler pipeline. As a direct implication, intermediate results are more easily accessible, and the generalization potential of the method is enhanced. The overall structure of WND-CHARM is conserved in CP-CHARM, while its building blocks have been modified: the underlying feature vector is built following CHARM’s structure, dimension reduction and classification are carried out using PCA-LDA, and testing is performed through 10-fold cross-validation. We also demonstrated that CP-CHARM could achieve similar performance as WND-CHARM, and further illustrated the practical use of our approach on other biological image sets, including some with more subtle phenotypes than in the original datasets. In our experiments, we highlighted and discussed possible batch effects and how to avoid them.

We close with a remark regarding the use of machine learning approaches in bioimaging. It is important to note that even using an appropriately designed reference image set where batch-related artifacts have been handled, whole-image-based methods still do not guarantee separation of classes to be based on biological mechanisms of interest. As an example, a biologist might train the system to successfully recognize a phenotype induced by a chemical compound of interest as compared to a negative control, but this recognition might entirely be based on the chemical simply slowing the cells’ growth rate. Because a slow growth rate yields more sparse cells in the images, the classifier may recognize this rather general phenotype rather than the specific cellular appearance caused by the chemical and of interest to the biologist. In such a case, including positive and negative control samples with varying cell density could mitigate this issue.

Therefore, care should be taken, not just to reduce the impact of potential batch-related artifacts, but also to interpret the classification results using biological common sense.

## Availability and requirements

- **Project name:** CP-CHARM
- **Project home page:**https://github.com/CellProfiler/CPCharm
- **Operating system(s):** Platform independent
- **Programming language:** Python
- **License:** BSD 3-Clause License

### Availability of supporting data

CP-CHARM code is freely available at [33]. It includes pipelines for feature extraction which can be used with CellProfiler release 2.1.0 [36], the code to execute the classifier, a user manual, and a complete runnable example. The classifier and the CellProfiler modules are implemented in Python and released under BSD 3-Clause license. The image databases used in this paper are available for download from the original provider’s site.

## Additional file

Additional file 1
**Supplementary documentation.** Supplementary text and figures. Additional information about the composition of the original CHARM feature vector, the integration of CP-CHARM in CellProfiler, and the different validation methods considered in the paper. (PDF 1167 kb)
