# Supplementary material for: CP-CHARM: segmentation-free image classification made accessible
Source: BMC Bioinformatics. 2016 Jan 27;17:51. doi: 10.1186/s12859-016-0895-y (PMC4729047; doi:10.1186/s12859-016-0895-y)
Supplement: Additional file 1 — Supplementary documentation. Supplementary text and figures. Additional information about the composition of the original CHARM feature vector, the integration of CP-CHARM in CellProfiler, and the different validation methods considered in the paper. (PDF 1167 kb) [file 12859_2016_895_MOESM1_ESM.pdf]

CP-CHARM: Segmentation-free image classification made accessible
Supplementary documentation

Virginie Uhlmann, Shantanu Singh and Anne E. Carpenter

September 2015

1 Composition of the CHARM feature vector

High Contrast Features			Polynomial decompositions			Pixel statistics			Textures	
Edge Statistics (28)	Gabor Features (7)	Image Statistics (34)	Chebyshev Statistics (32)	Chebyshev-Fourier Statistics (32)	Zernike Polynomials † (72)	Moments * (4)	Multiscale Histogram * (24)	Radon Transform Statistics # (12)	Haralick Texture * (28)	Tamura Textures * (6)
• Mean<br>• Median<br>• Variance<br>• 8-bins histogram<br>• Number of edge pixels<br>• Direction Homogeneity<br>• Edge direction difference (4-bins histogram)	• Gabor features for f_0=[1…7]	• Euler number<br>• Centroids (x and y)<br>• Min<br>• Max<br>• Mean<br>• Variance<br>• Median<br>• 10-bins histogram<br>• Distance from object centroid to image centroid	• 32-bins histograms of the 400 coefficients of the Chebyshev Transform of the image	• Modulus of the complex coefficient of the Fourier Transform of the Chebyshev Transform of the image	• Modulus of the Zernike Features (i.e. the coefficients of the Zernike approximation of the image)	• Mean (1st)<br>• Variance (2nd)<br>• Skewness (3rd)<br>• Kurtosis (4th)	• 3-bins histogram<br>• 5-bins histogram<br>• 7-bins histogram<br>• 9-bins histogram	• 3-bins histogram at 0°<br>• 3-bins histogram at 45°<br>• 3-bins histogram at 90°<br>• 3-bins histogram at 135°	• Statistics based on the co-occurrence matrix of the image	• Contrast<br>• Coarseness<br>• Directionality<br>• 3-bins histogram of coarseness

† = features computed both on the original image and on its Fourier Transform
# = features computed on the original image, on its Fourier Transform, on its Chebyshev Transform, and on the Chebyshev Transform of its Fourier Transform
* = features computed on the original image, on its Wavelet Transform, on its Chebyshev Transform, on its Fourier Transform, on the Wavelet Transform of its Fourier Transform, and on the Chebyshev Transform of its Fourier Transform

Figure S1: **Composition of the CHARM vector as proposed by [1].** The different feature groups are highlighted in the top row. Features extracted on higher image levels are denoted by a dagger (†), star (∗) and a sharp (#).

The *CHARM* (Compound Hierarchy of Algorithms Representing Morphology) vector is composed of 1025 elements extracted from the original image, as well as from transforms of the image. This large collection of measurements fall into four main categories, which are further composed of several measurement sets: high contrast features, polynomial decompositions, pixel statistics, and textures, all extracted from gray-scale images. The high contrast features category contains information about the elements that compose the image, such as edges and shapes. The textures

category contains some well-known texture descriptors such as the Haralick [2] and the Tamura [3] texture features. The pixel statistics group is composed of information about pixel values distribution over the image, such as histograms with various numbers of bins and statistical moments. Finally, the polynomial decompositions category is built by generating a polynomial that approximates pixel values up to a given error such as the Zernike or the Chebyshev polynomials, and whose coefficients describe the image. Some of these features are extracted from the original image as well as from transforms and from compound transforms (transforms of transforms) of the image. The Fourier transform, Wavelet transform, Chebyshev transform and composition of these transforms are used to give additional higher level interpretation of the image content, for instance, in the case of the Fourier transform, by revealing the frequency composition of the image. The construction of the CHARM vector is illustrated in Figure S1. A formal definition of each of these elements along with appropriate references is given in [1].

2 Integration in CellProfiler

The feature extraction step of CP-CHARM is carried out in CellProfiler [4], as depicted in Figure S2. The 953 measurements required to build the feature vector are extracted through a user-friendly "pipeline" composed of different modules. This construction offers greater modularity and flexibility: the user is able to easily tune the feature vector content, which would not be possible using the original WND-CHARM implementation without significant programming expertise. Here, the removal of existing modules, the introduction of new ones or the modification of existing modules settings in the pipeline is made easy through CellProfiler's interface, as depicted in Figure S2.

Most features extracted by our method require pixel-based computations, which can be computationally expensive for large-scale images. As feature extraction is performed in CellProfiler, which allows for efficient batch processing, one way of dealing with such data is to tile them into smaller chunks to be processed in batch mode. The final classification result for the large image can then be retrieved as the most represented class among classification results from the tiles, or as a percentage of the tiles.

3 Study of validation methods

Using our Python implementation of WND-CHARM, which has been shown to give similar results as the published C++ version [5], we performed repeated rounds of training and testing on WND-CHARM's reference suite. As choosing a different validation method than what was initially proposed

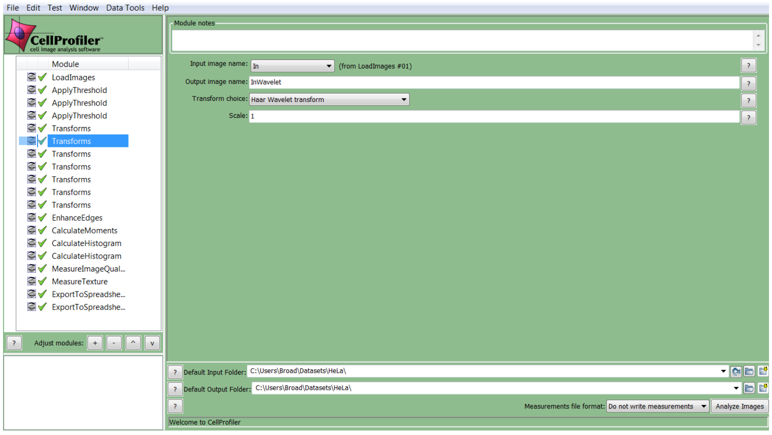

Figure S2: **CellProfiler user interface with an example pipeline composed of several modules.** Each module contains a description of its parameters, which can be modified through the interface. Modules can easily be added to or removed from the pipeline.

in WND-CHARM had a direct impact on the measure of performance of the algorithm, we investigated the effect of changing WND-CHARM's validation method while keeping the rest of the algorithmic structure unchanged.

First, we gathered results using WND-CHARM's original custom validation method, which we refer to as lone 4-fold cross-validation. Then, in the same experimental conditions, we changed the validation method for 10-fold cross-validation while keeping all other parts of the algorithm unaltered. The results on WND-CHARM's reference suite, presented in Table S1, show that similar median classification accuracies are obtained with either validation method. Using 10-fold cross-validation, we however observe much smaller standard deviations, implying that classification results are more stable over the different repetitions of the experiment. Considering N rounds of training and testing, this can easily be explained from the fact that 10-fold cross-validations results are subject to two steps of averaging. They correspond to averages of N results, which are themselves obtained by averaging classification results over each of the possible combinations of 10 given folds. Conversely, lone 4-fold cross-validation results are obtained by direct averaging of N classification results for a particular $\frac{3}{4}$ versus $\frac{1}{4}$ partitions of the data into training and testing sets. The outcome of this experiment allows us to safely further compare results obtained with WND-CHARM and CP-CHARM, at least regarding median classification accuracy, even though their validation methods differ.

Table S1: Comparison of validation methods using WND CHARM's reference suite

	**Lone 4-fold cross-validation**		**10-fold cross-validation**	
Dataset	Median	Std Dev.	Median	Std Dev.
AT&T	0.97	0.02	0.97	6e-3
Brodatz	0.91	0.01	0.93	5e-3
CHO	0.93	0.02	0.94	1e-3
COIL-20	1.00	1e-3	0.99	3e-4
HeLa	0.87	0.09	0.85	6e-3
Pollen	0.95	0.02	0.96	4e-3
Yale	0.83	0.07	0.79	0.03

Note: 1.0 corresponds to 100% correct classification.

4 Supplementary Figures

Figure S3: Misclassification rates per non-overlapping feature "groups".

Figure S4: Misclassification rates per non-overlapping feature "levels".

Figure S5: Feature group composition and weights in the top 15% subset retained by WND-CHARMs feature selection method.

Figure S6: Tanimoto distance matrices measuring the similarity of the top features subsets content across 100 classification runs of the original WND-CHARM algorithm on the reference datasets.

Figure S7: Misclassification rates over 10 runs of training and testing using the CHARM-like feature vector and classifying with methods allowing for non-linearites or not.

Figure S8: Misclassification rates over 10 runs of training and testing using the CHARM-like feature vector and different classifiers.

References

[1] Orlov, N., Shamir, L., Macura, T., Johnston, J., Eckley, D.M., Goldberg, I.G.: Wnd-charm: Multi-purpose image classification using compound image transforms. Pattern recognition letters **29**(11), 1684–1693 (2008)

[2] Haralick, R.M., Shanmugam, K., Dinstein, I.: Textural features for image classification. IEEE Transactions on Systems, Man and Cybernetics (6), 610–621 (1973)

[3] Tamura, H., Mori, S., Yamawaki, T.: Textural features corresponding to visual perception. IEEE Transactions on Systems, Man and Cybernetics **8**(6), 460–473 (1978)

[4] Carpenter, A.E., Jones, T.R., Lamprecht, M.R., Clarke, C., Kang, I.H., Friman, O., Guertin, D.A., Chang, J.H., Lindquist, R.A., Moffat, J., Golland, P., Sabatini, D.M.: Cellprofiler: image analysis software for identifying and quantifying cell phenotypes. Genome biology **7**(10), 100 (2006)

[5] Uhlmann, V.: A segmentation-free image classifier for biological applications. Master's thesis, Swiss Federal Institute of Technology, Lausanne (EPFL) (2012)

(a)

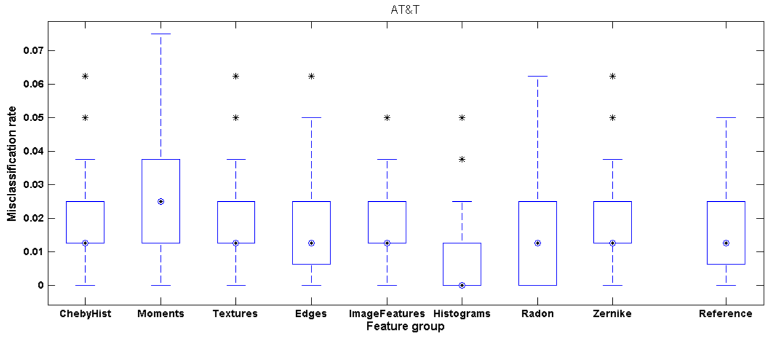

(b)

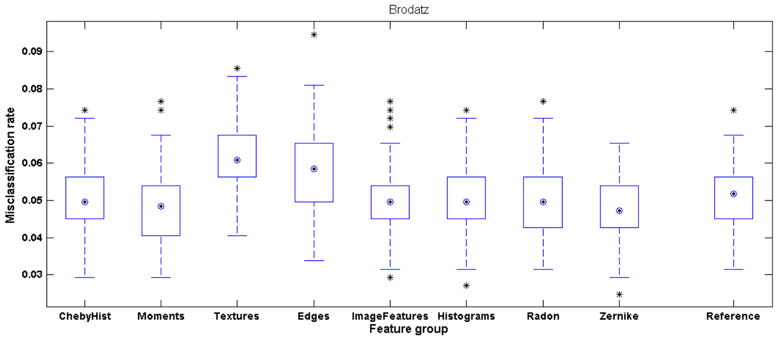

(c)

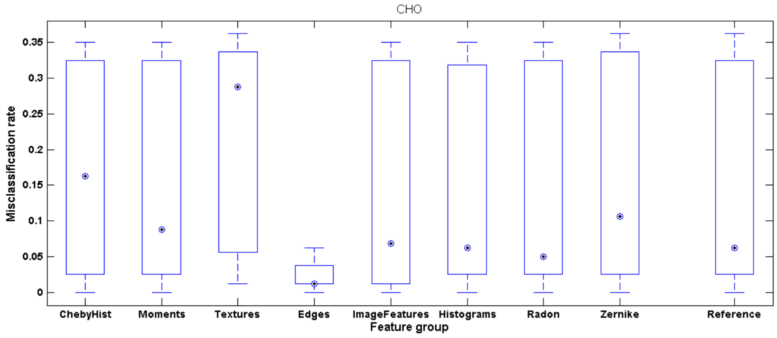

Figure S3: **Misclassification rates per non-overlapping feature "groups".** To be continued on next page.

(d)

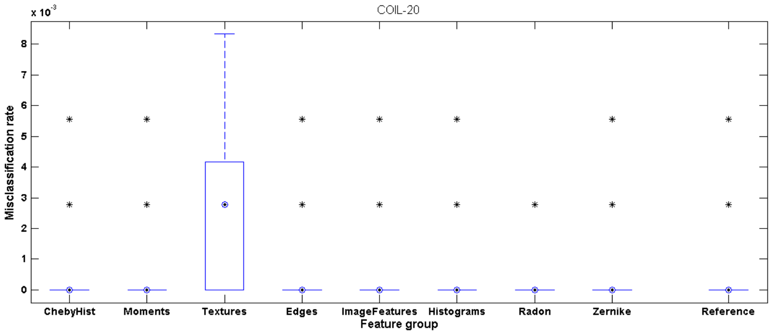

(e)

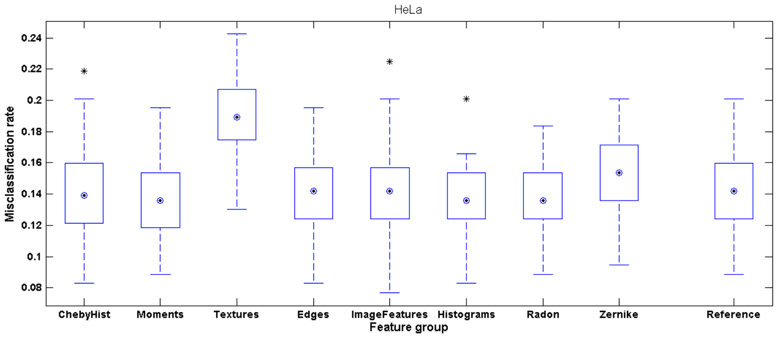

(f)

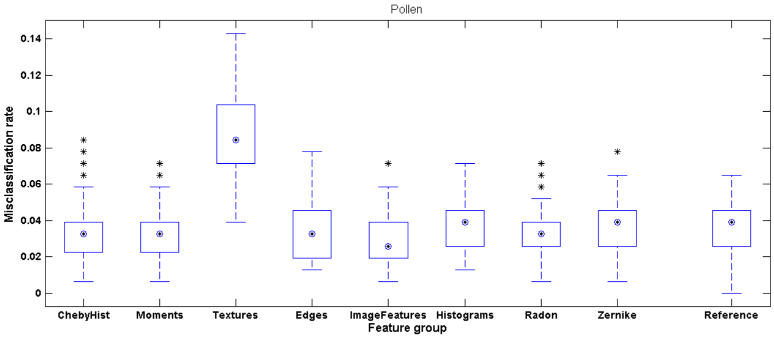

Figure S3: **Misclassification rates per non-overlapping feature "groups".** To be continued on next page.

(g)

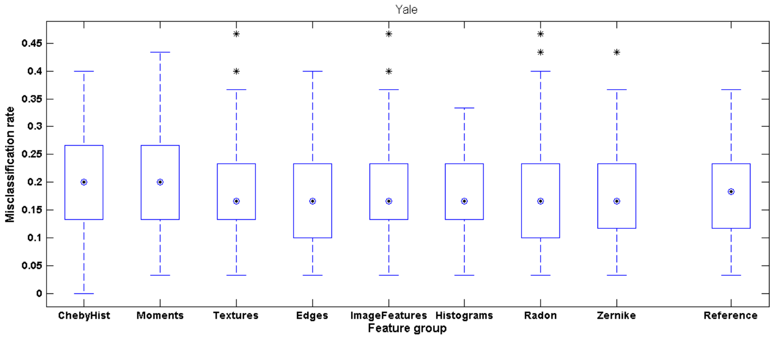

Figure S3: **Misclassification rates per non-overlapping feature "groups".** The label under each bar corresponds to the group that has been removed from the CHARM vector for classification using WND (each element listed in Figure S1 belongs to one of the groups). The rightmost box corresponds to the reference classification accuracy distribution using the whole feature set. Results range from 0 (0%) to 1 (100%) and were obtained over 10 runs of training and testing using 10-fold cross-validation. (a) AT&T, (b) Brodatz, (c) CHO, (d) COIL-20, (e) HeLa, (f) Pollen, (g) Yale. We note that COIL-20 poses a significantly simpler classification problem compared to other datasets; there is very little variation in the misclassification rates when dropping any single group of features, except for the Textures group.

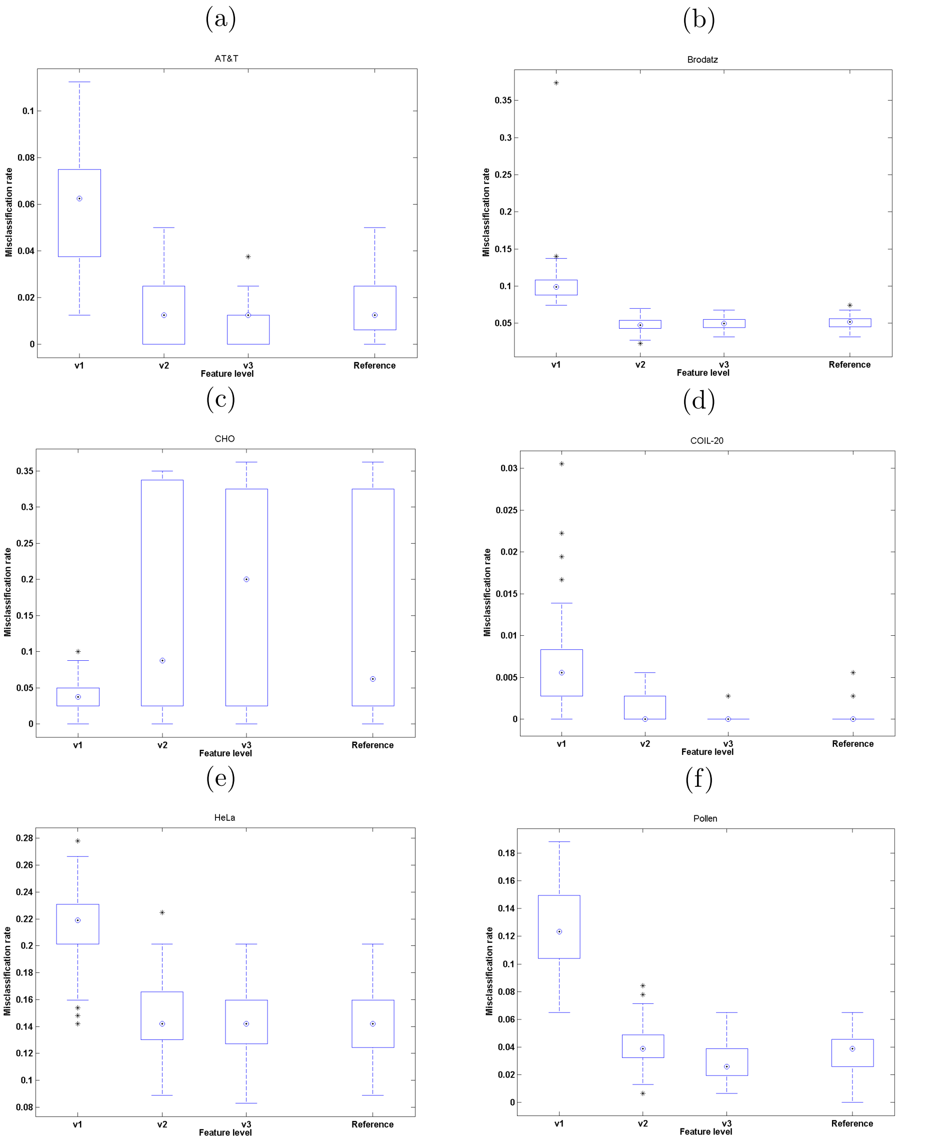

Figure S4: **Misclassification rates per non-overlapping feature "levels".** To be continued on next page.

(g)

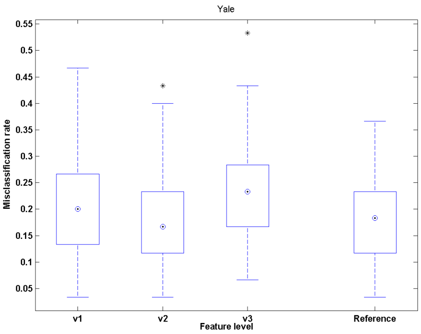

Figure S4: **Misclassification rates per non-overlapping feature "levels".** The label under each bar corresponds to the level that has been removed from the CHARM vector for classification using WND (v1: features extracted from the original image only, v2: features extracted from transforms of the image, v3: features extracted from transforms of transforms of the image). The rightmost box corresponds to the reference classification accuracy distribution using the whole feature set. All results range from 0 (0%) to 1 (100%) and were obtained over 10 runs of training and testing using 10-fold cross-validation. (a) AT&T, (b) Brodatz, (c) CHO, (d) COIL-20, (e) HeLa, (f) Pollen, (g) Yale.

(a)

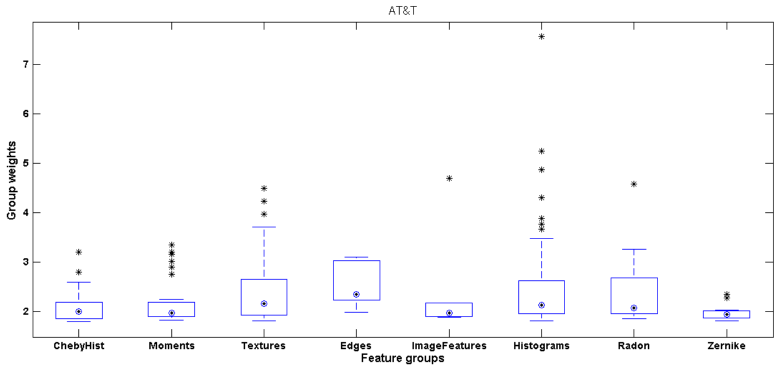

(b)

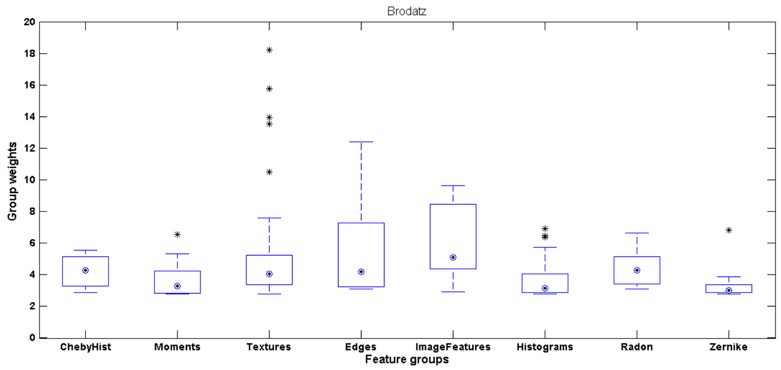

(c)

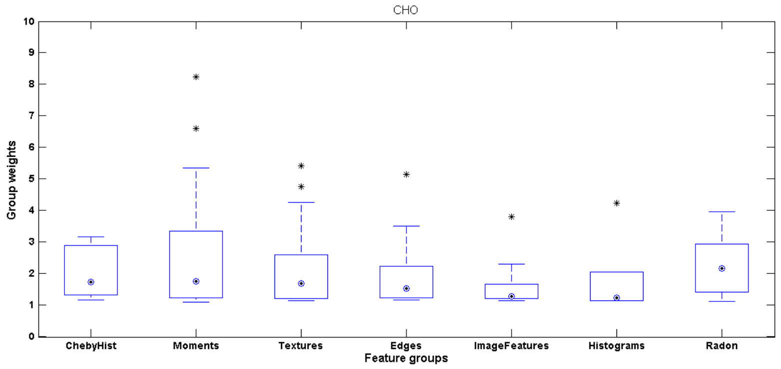

Figure S5: **Feature group composition and weights in the top 15% subset retained by WND-CHARMs feature selection method.** To be continued on next page.

(d)

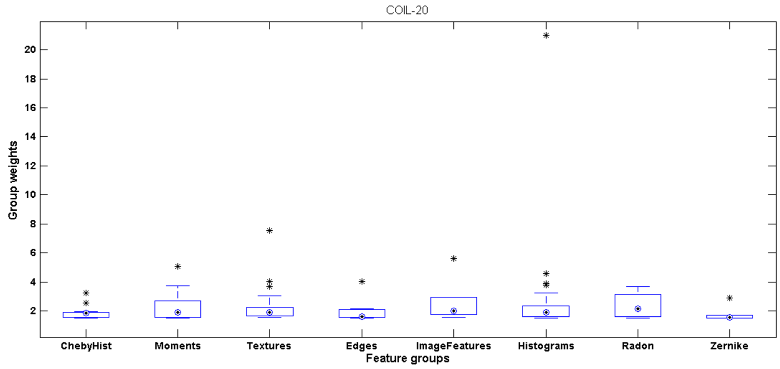

(e)

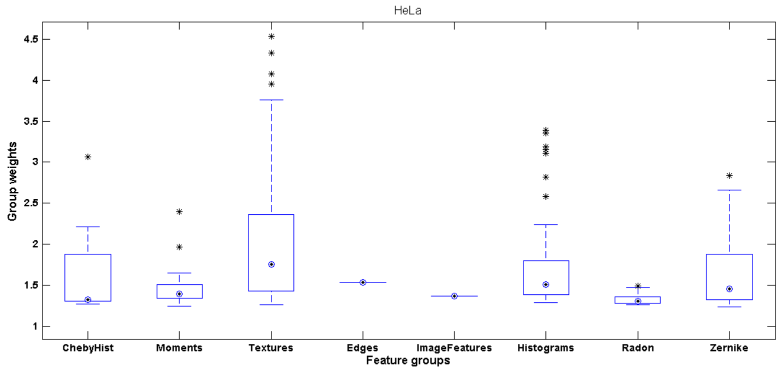

(f)

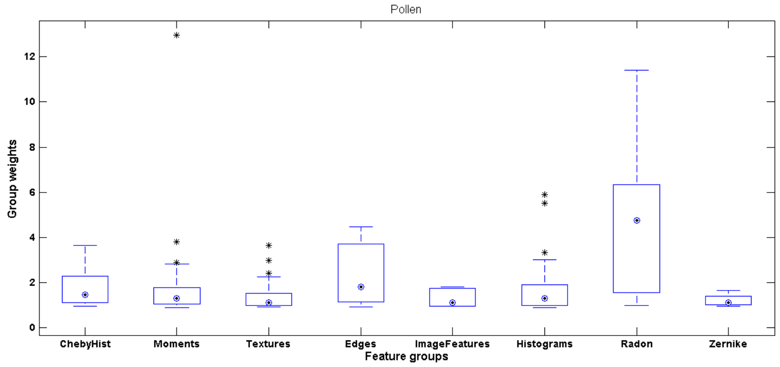

Figure S5: **Feature group composition and weights in the top 15% subset retained by WND-CHARMs feature selection method.** To be continued on next page.

(g)

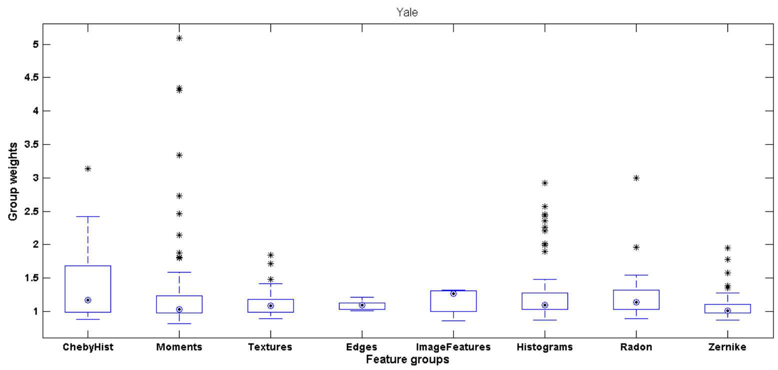

Figure S5: **Feature group composition and weights in the top 15% subset retained by WND-CHARMs feature selection method.** High weights indicate features identified as having a large discriminative power by WND-CHARMs weighting scheme. (a) AT&T, (b) Brodatz, (c) CHO, (d) COIL-20, (e) HeLa, (f) Pollen, (g) Yale. This experiment highlights groups that are important for classification (as they get strong weights), while Figure S3 shows misclassification rates when datasets are being classified using a feature vector version where one particular group (the one labeled below each boxplot) has been removed from the feature vector. One would expect a group with strong weights in the above plots to significantly affect misclassification rate when removed (which could be observed in Figure S3). In some cases, the removal of feature groups containing strong weights indeed impacts classification accuracy (*e.g.*, the Textures group in (e), as seen in Figure S3e). In some other situations, however, feature groups collecting strong weights do not seem to play an important role in the final classification result (*e.g.*, the Radon group in (f), as seen in Figure S3f).

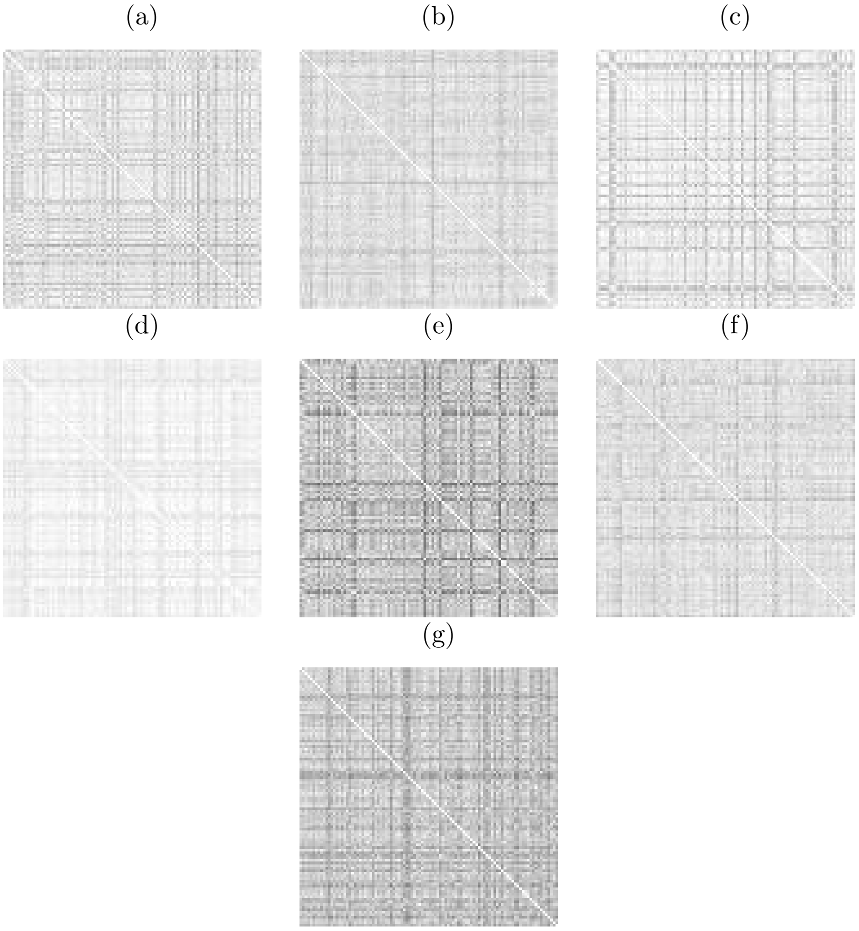

Figure S6: **Tanimoto distance matrices measuring the similarity of the top features subsets content across 100 classification runs of the original WND-CHARM algorithm on the reference datasets.** The Tanimoto distance is computed for every pairs of subsets. Since our data covered 100 runs of classification, results are summarized in 100×100 pixels matrices. There, black corresponds to a Tanimoto distance of 0 and white to 1. A perfectly stable feature selection scheme would yield an all-white matrix as a value of one indicates perfectly similar subsets. (a) AT&T, (b) Brodatz, (c) CHO, (d) COIL-20, (e) HeLa, (f) Pollen, (g) Yale.

(a)

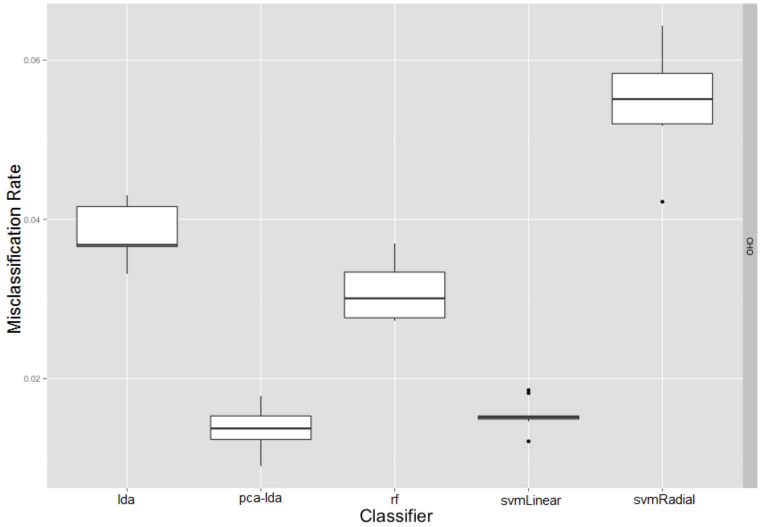

(b)

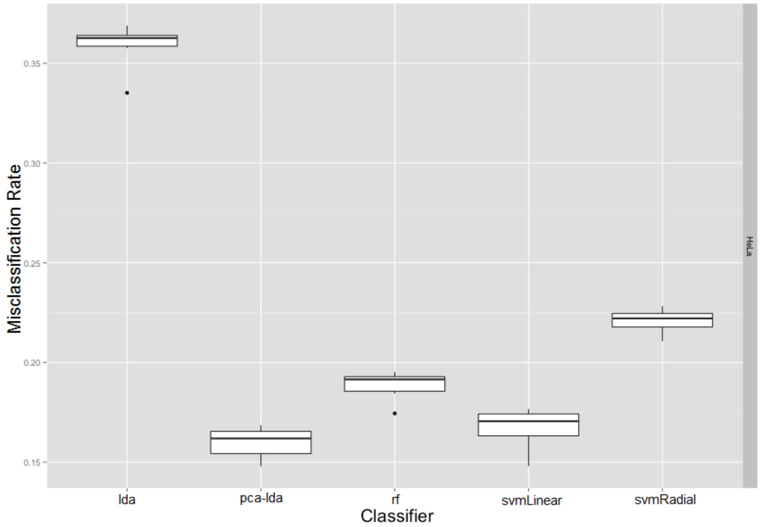

Figure S7: **Misclassification rates over** 10 **runs of training and testing using the CHARM-like feature vector and classifying with methods allowing for non-linearites or not.** To be continued on next page.

(c)

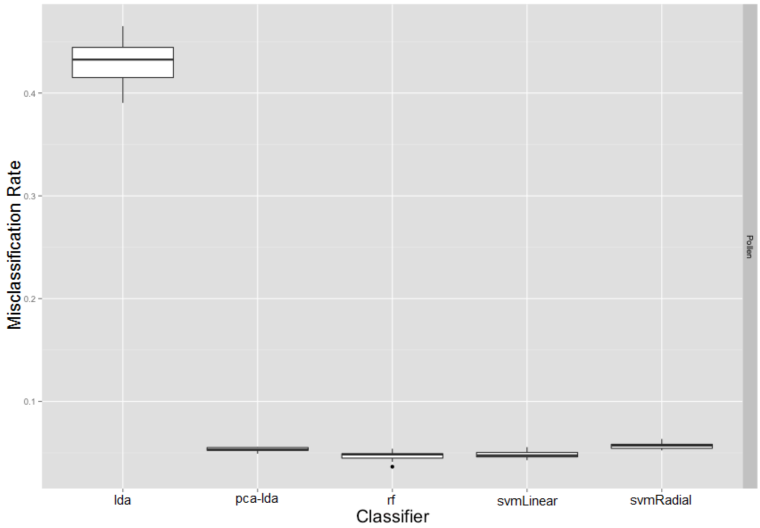

(d)

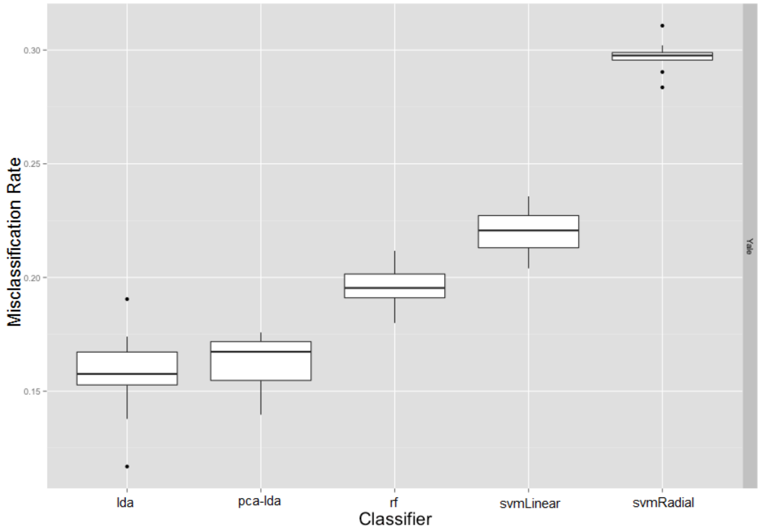

Figure S7: **Misclassification rates over** 10 **runs of training and testing using the CHARM-like feature vector and classifying with methods allowing for non-linearites or not.** From left to right: LDA, PCA-LDA, random forests, linear SVM and radial basis functions SVM, using 10-fold cross-validation. LDA, PCA-LDA and linear SVM are purely linear methods, while random forests and radial basis functions SVM allow for nonlinearities. Results range from 0 (0%) to 1 (100%). (a) CHO, (b) HeLa, (c) Pollen and (d) Yale reference datasets. Results suggest that linearly separability is a reasonable assumption in the CP-CHARM feature space for WND-CHARMs reference suite datasets, as allowing for nonlinearities do not improve classification performance.

(a)

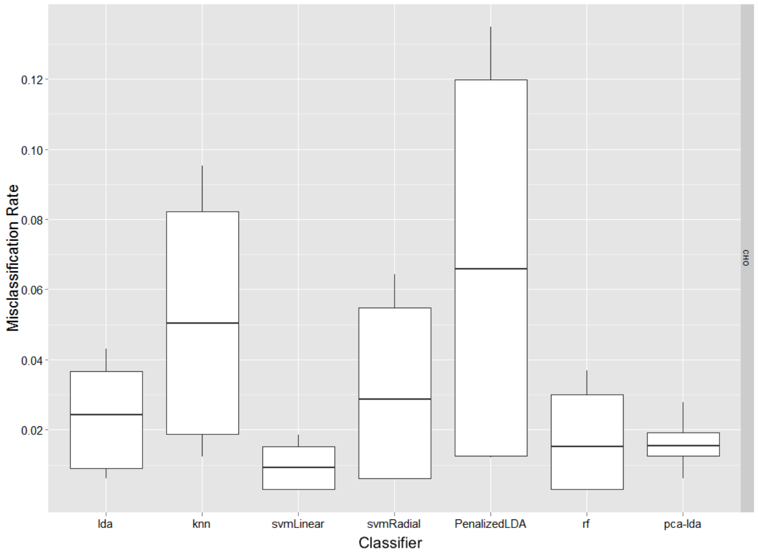

(b)

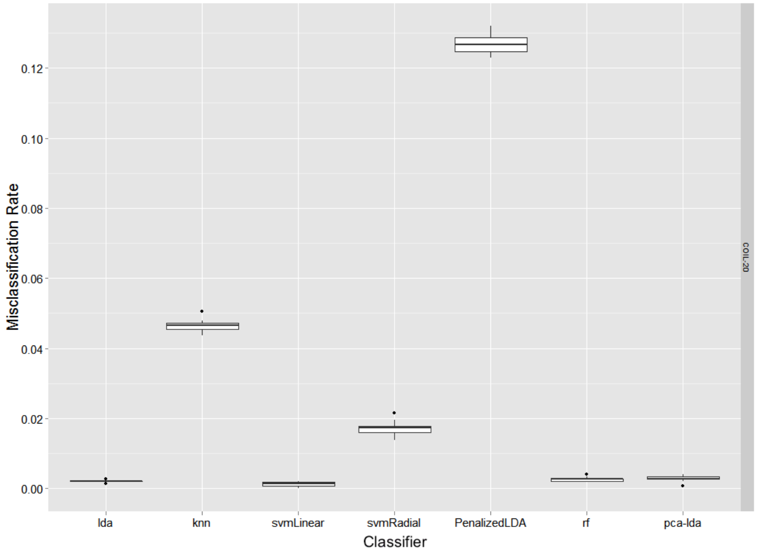

Figure S8: **Misclassification rates over 10 runs of training and testing using the CHARM-like feature vector and different classifiers.** To be continued on next page.

(c)

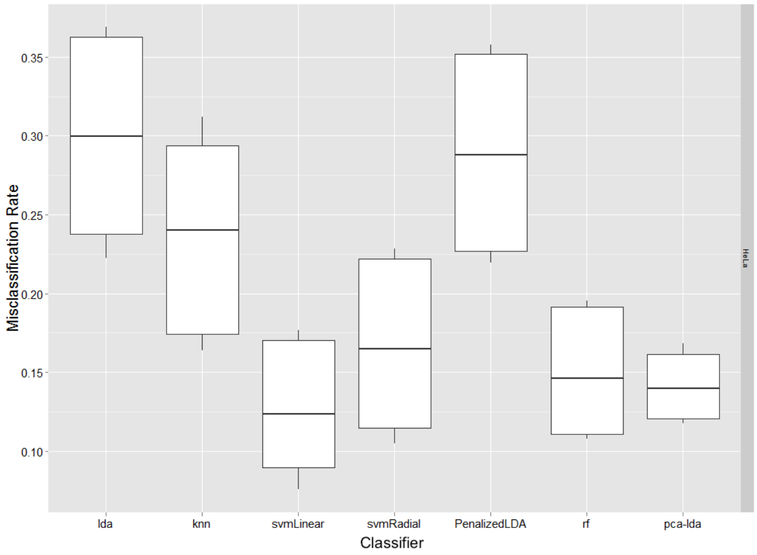

(d)

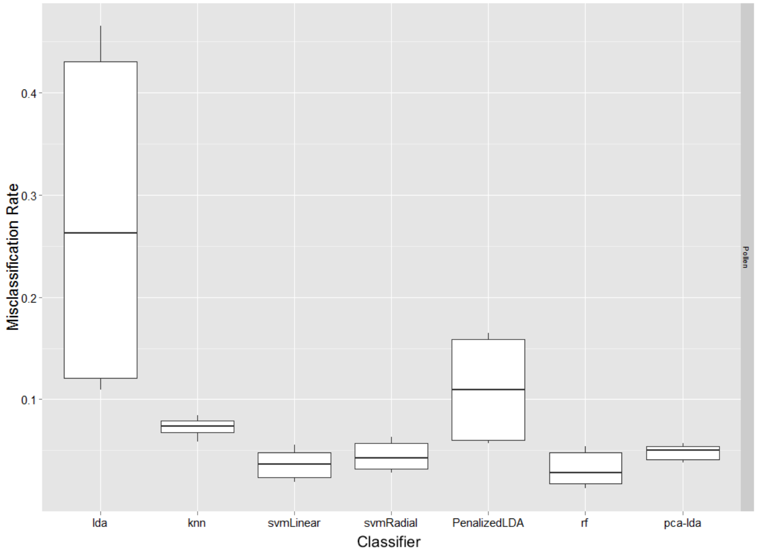

Figure S8: **Misclassification rates over 10 runs of training and testing using the CHARM-like feature vector and different classifiers.** To be continued on next page.

(e)

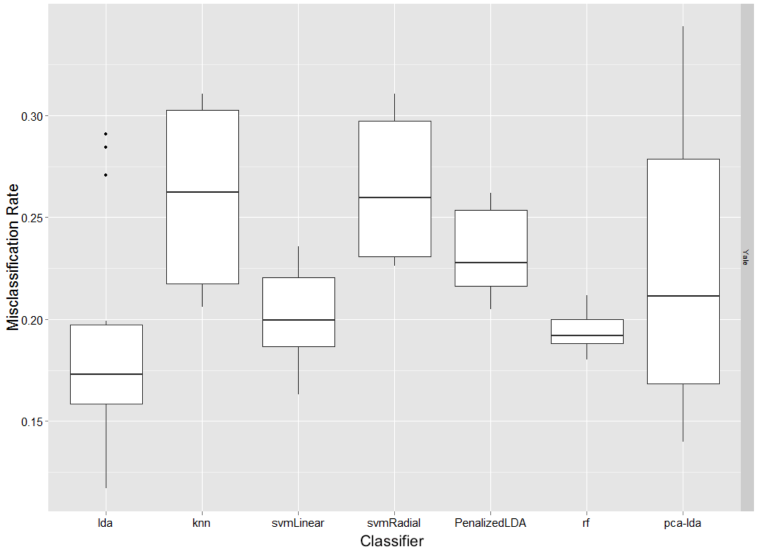

Figure S8: **Misclassification rates over** 10 **runs of training and testing using the CHARM-like feature vector and different classifiers.** From left to right: LDA, kNN, linear SVM, radial basis functions SVM, penalized LDA, random forests and PCA-LDA, using 10-fold cross-validation. Results range from 0 (0%) to 1 (100%). (a) CHO, (b) COIL-20, (c) HeLa, (d) Pollen, (e) Yale.
